# MqsR/MqsA Toxin/Antitoxin System Regulates Persistence and Biofilm Formation in *Pseudomonas putida* KT2440

**DOI:** 10.3389/fmicb.2017.00840

**Published:** 2017-05-09

**Authors:** Chenglong Sun, Yunxue Guo, Kaihao Tang, Zhongling Wen, Baiyuan Li, Zhenshun Zeng, Xiaoxue Wang

**Affiliations:** ^1^Key Laboratory of Tropical Marine Bio-resources and Ecology, Guangdong Key Laboratory of Marine Materia Medica, RNAM Center for Marine Microbiology, South China Sea Institute of Oceanology, Chinese Academy of SciencesGuangzhou, China; ^2^University of Chinese Academy of SciencesBeijing, China

**Keywords:** toxin/antitoxin system, MqsR/MqsA, biofilm, persistence, *Pseudomonas putida*

## Abstract

Bacterial toxin/antitoxin (TA) systems have received increasing attention due to their prevalence, diverse structures, and important physiological functions. In this study, we identified and characterized a type II TA system in a soil bacterium *Pseudomonas putida* KT2440. This TA system belongs to the MqsR/MqsA family. We found that PP_4205 (MqsR) greatly inhibits cell growth in *P. putida* KT2440 and *Escherichia coli*, the antitoxin PP_4204 (MqsA) neutralizes the toxicity of the toxin MqsR, and the two genes encoding them are co-transcribed. MqsR and MqsA interact with each other directly *in vivo* and MqsA is a negative regulator of the TA operon through binding to the promoter. Consistent with the MqsR/MqsA pair in *E. coli*, the binding of the toxin MqsR to MqsA inhibits the DNA binding ability of MqsA in *P. putida* KT2440. Disruption of the *mqsA* gene which induces *mqsR* expression increases persister cell formation 53-fold, while overexpressing *mqsA* which represses *mqsR* expression reduces persister cell formation 220-fold, suggesting an important role of MqsR in persistence in *P. putida* KT2440. Furthermore, both MqsR and MqsA promote biofilm formation. As a DNA binding protein, MqsA can also negatively regulate an ECF sigma factor AlgU and a universal stress protein PP_3288. Thus, we revealed an important regulatory role of MqsR/MqsA in persistence and biofilm formation in *P. putida* KT2440.

## Introduction

Toxin/antitoxin (TA) systems were initially discovered in 1983 as plasmid addiction systems on low-copy-number plasmids due to their ability to stabilize plasmids in the bacterial host population (Ogura and Hiraga, 1983). After that, thousands of TA loci have been identified on plasmids, prophages as well as on the chromosomes of bacteria and archaea (Pandey and Gerdes, 2005; Yamaguchi et al., 2011). TA systems have received increasing attention due to their prevalence, diverse structures, and important physiological functions. In the last decade, TA systems in bacterial chromosomes have been extensively studied. The roles of TA systems in cell physiology, specifically in biofilm formation, persister cell formation (Dorr et al., 2010; Kim and Wood, 2010), the general stress response and phage inhibition are becoming clearer (Pecota and Wood, 1996; Shah et al., 2006; Magnuson, 2007; Tsilibaris et al., 2007; Kim et al., 2009). Till now, TA systems have been classified in to six different groups (Types I–VI) according to the nature of the antitoxin and the mode of interaction between the toxin and antitoxin (Page and Peti, 2016). About 40 TA systems have been reported in *Escherichia coli*, including 18 type I, 19 type II, 1 type IV, 1 type V, and 1 type VI (Baba et al., 2006; Yamaguchi and Inouye, 2011; Masuda et al., 2012; Wang et al., 2012; Aakre et al., 2013; Guo et al., 2014). More than 88 type II TAs have been identified in human pathogen *Mycobacterium tuberculosis* (Winther et al., 2016).

The most-studied chromosomal encoded TA systems include MazE/MazF, RelB/RelE, MqsR/MqsA, HigB/HigA, and VapC/VapB which belong to the type II TA systems. Toxins of these TA systems function as endoribonucleases, and regulate protein production by selectively degrading RNAs (Wang and Wood, 2011). Physiological functions of some of these type II toxins are associated with persister cell formation and biofilm formation. It has been reported that persister cell formation was reduced after deleting 10 toxins of the type II TA loci in *E. coli* (Maisonneuve et al., 2011). An earlier study showed that *mqsR* was the most highly induced gene in persister cells compared to non-persisters (Shah et al., 2006). The MqsR/MqsA TA system in *E. coli* has been shown to regulate biofilm formation (Barrios et al., 2006; Kim et al., 2010; Wang et al., 2011; Hong et al., 2012). MqsR/MqsA pair of *E. coli* is the first TA linked to biofilm formation since *mqsR* was induced in a transcriptome study that identified genes that were differentially regulated in biofilm cells (Ren et al., 2004). MqsR encodes a ribosome-independent that cleaves mRNA primarily at GCU sites (Yamaguchi et al., 2009; Christensen-Dalsgaard et al., 2010). Overproduction of MqsR led to a global change in the transcriptome profile due to its mRNA interferase activity which has substrate specificity for cellular mRNAs (Kim et al., 2010). Specifically, 14 mRNAs that lack a GCU site (Yamaguchi et al., 2009) were enriched when MqsR was activated including a novel type I toxin RalR (Guo et al., 2014) and the first reported Type V toxin GhoT (Wang et al., 2012). Six out of these 14 mRNAs were differentially regulated in biofilm (Domka et al., 2007). Moreover, antitoxin MqsA represses the expression of stationary sigma factor *rpoS* which reduces the concentration of the internal messenger c-di-GMP and lead to increased motility and decreased biofilm formation in *E. coli* (Wang et al., 2011). Thus, both MqsR and MqsA play an important role in *E. coli* biofilm formation.

Many *Pseudomonas* are known for their adaptability to thrive in diverse and even hostile habitats (Silby et al., 2011). *Pseudomonas putida* is an archetypical environmental microbe and is commonly found in various soil and aquatic environments including those were heavily polluted (Ramos et al., 2009). *P. putida* strain KT2440 genome is fully sequenced, and it serves as a standard reference to compare with other *P. putida* strains and with pathogenic *Pseudomonas aeruginosa* strains (Dos Santos et al., 2004). *P. putida* and *P. aeruginosa* are closely related and share approximately 85% of the predicted coding regions, but *P. putida* lacks the key virulence genes such as the type III secretion systems (Nelson et al., 2002). Genomic analysis of *P. putida* KT2440 also revealed that the high diversity of mobile elements, extracytoplasmatic function (ECF) sigma factors and stress response regulators are indicative of its renowned tolerance of environmental stresses and adaptation to diverse habitats (Dos Santos et al., 2004). TA systems may also contribute to the stress tolerance of *P. putida* living in heavily polluted environments through regulating biofilm formation and persistence. The first TA system characterized in pseudomonads was the type II TA system GraT/GraA of *P. putida* KT2440 which belong to the HigB/HigA TA family (Tamman et al., 2014). GraT of the GraT/GraA system exhibited moderate toxin at optimal growth temperatures but caused a severe growth defect at lower temperatures (Tamman et al., 2014). Recent work demonstrated that GraT toxin can also increase resistance to different antibiotics (Tamman et al., 2014), and inhibit ribosome biogenesis at low temperatures by interacting with the DnaK chaperone (Ainelo et al., 2016). Recently study of the HigB/HigA TA system in *P. aeruginosa* showed that the activation of HigB reduced the production of virulence factors and biofilm formation (Wood and Wood, 2016), while another study showed the activation of HigB in *P. aeruginosa* increased type III secretion system-mediated cytotoxicity (Li et al., 2016).

Besides *E. coli*, MqsR/MqsA TA system is distributed in a large number of genera such as *Pseudomonas, Yesinia, Burkholderia*, and *Xylella* (Makarova et al., 2009). Recent work showed that in plant pathogen *Xylella fastidiosa*, overexpressing the MqsR homolog increased persister cell formation and biofilm formation while repressed cell motility (Merfa et al., 2016). PP_4205/PP_4204 in *P. putida* KT2440 share medium identity (∼50%) with the MqsR/MqsA in *E. coli* K-12, however, the function of MqsR/MqsA in *Pseudomonas* remains unknown. Our previous work showed that MqsR variants not only exhibit different toxicity but also exhibit different ability to induce persister formation in *E. coli* (Hong et al., 2012). Thus, to explore the role of MqsR and MqsA homologs in different bacterial hosts would help gain more insights into the prevalence of TA system and how they participate in bacterial stress response in different hosts.

In this study, we first characterized PP_4205 and PP_4204 and further explored their function in *P. putida* KT2440. The two genes encoding MqsR homolog and MqsA homolog are co-transcribed and both encode small proteins. MqsR and MqsA interact with each other directly *in vivo* and MqsA is a negative regulator of the TA operon through binding to the promoter. Consistent with the MqsR/MqsA pair in *E. coli*, the MqsR inhibits the binding of MqsA to its promoter in *P. putida* KT2440. Disruption of the *mqsA* gene which induces *mqsR* expression increases persister cell formation while overexpressing *mqsA* which represses *mqsR* expression reduces persister cell formation, suggesting an important role of MqsR in persistence in *P. putida* KT2440. However, different from *E. coli*, both MqsR and MqsA promote biofilm formation in *P. putida* KT2440. As a DNA binding protein, MqsA in *E. coli* also regulates the stationary phase sigma factor RpoS and CsgD. In contrast, we found that MqsA negatively regulates the ECF sigma factor AlgU and a universal stress protein PP_3288 in *P. putida* KT2440, suggesting a different role of MqsR/MqsA in *P. putida* KT2440.

## Materials and Methods

### Bacterial Strains, Plasmids, and Growth Conditions

The bacterial strains and plasmids used in this study are listed in **Table 1**, and the sequences of the primers are listed in Supplementary Table S1. The *E. coli* and *P. putida* KT2440 strains were grown in Luria Bertani broth (LB) medium at 30°C except where indicated. Gentamicin (15 μg/ml) was used for pre-culturing isogenic knockout mutants and for maintaining pEX18Ap-based plasmids, carbenicillin (100 μg/ml) was used to maintain plasmid pMQ70, and kanamycin (50 μg/ml) was used to maintain plasmids pET28b, pHGE, and pHGR01.

**Table 1:** Bacterial strains and plasmids used in this study.

Strains/Plasmids	Genotype or description	Source
***P. putida* KT2440**		
Wild-type	Prototroph	Stover et al., 2000
Δ*mqsR*	Replacement of *mqsR* gene by Gm^R^-encoding cassette in KT2440	This study
Δ*mqsRA*	Replacement of *mqsR* and *mqsA* genes by Gm^R^-encoding cassette in KT2440	This study
Δ*mqsA*	Replacement of *mqsA* gene by Gm^R^-encoding cassette in KT2440	This study
***E. coli* strains**		
BL21(DE3)	F*^-^ompT hsdS_B_(r_B_*^-^*m_B_*^-^*) gal dcm λ*(DE3) Ω P_tacUV 5_::T7 polymerase	Novagen
WM3064	*thrB*1004 *pro thi rpsL hsdS lacZ*ΔM15 RP4-1360 Δ(*araBAD*)567 Δ*dapA*1341::[*erm pir*(wt)]	W. Metcalf, UIUC
**Plasmids**		
pMQ70	Carbbb, Ampbbb, P*_BAD_* expression vector	Shanks et al., 2006
pMQ70-*mqsA*	Carbbb, Ampbbb, P*_BAD_*:: *mqsA*	This study
pMQ70-*mqsR*	Carbbb, Ampbbb, P*_BAD_*:: *mqsR*	This study
pMQ70-*mqsRA*	Carbbb, Ampbbb, P*_BAD_*:: *mqsR-mqsA*	This study
pHGE	pHGE-P*_tac_*, Kmbbb; IPTG inducible expression vector in *P. putida* KT2440	Invitrogen
pHGE-*mqsA*	Kmbbb; expression vector for *mqsA*	This study
pHGE-*mqsR*	Kmbbb; expression vector for *mqsR*	This study
pHGE-*mqsRA*	Kmbbb; expression vector for *mqsR-mqsA*	This study
pET28b	Kmbbb, *lacI*^q^, P*_T7_* expression vector	Novagen
pET28b-*mqsA*-His	Kmbbb, *lacI*^q^, pET28b P*_T7-lac_*:: *mqsA* with MqsA C-terminus His-tagged	This study
pET28b-*mqsRA*-CHis	Kmbbb, *lacI*^q^, pET28b P*_T7-lac_*:: *mqsR-mqsA* with MqsA C-terminus His-tagged	This study
pET28b-*mqsRA*	Kmbbb, *lacI*^q^, pET28b P*_T7-lac_*:: *mqsR-mqsA* without His-tag	This study
pEX18Ap	Ampbbb; *oriT*^+^ *sacB*^+^, gene replacement vector with MCS from pUC18	Hoang et al., 1998
pPS856	Ampbbb, Gm^R^; contains Gm^R^-encoding cassette	Hoang et al., 1998
pEX18Ap-Gm-*mqsA*	Ligation of a Gm fragment from pPS856 between homologous arm of *mqsA* in pEX18Ap	This study
pEX18Ap-Gm-*mqsRA*	Ligation of a Gm fragment from pPS856 between homologous arm of *mqsR-mqsA* in pEX18Ap	This study
pEX18Ap-Gm-*mqsR*	Ligation of a Gm fragment from pPS856 between homologous arm of *mqsR* in pEX18Ap	This study
pHGR01	Kmbbb, R6K *ori*, promoterless-*lacZ* reporter vector	Fu et al., 2014
pHGR01-P*mqsRA*-*lacZ*	Fuse *mqsRA* promoter with *lacZ* in pHGR01	This study
pHGR01-P*mqsRA-mqsA*-*lacZ*	Fuse *mqsRA* promoter, *mqsA* gene and *lacZ* in pHGR01	This study
pHGR01-P*algU*-*lacZ*	Fuse *algU* promoter with *lacZ* in pHGR01	This study
pHGR01-P*PP_3288*-*lacZ*	Fuse *PP_3288* promoter with *lacZ* in pHGR01	This study

*Car^R^, Amp^R^, Km^R^, and Gm^R^ indicate carbenicillin, ampicillin, kanamycin, and gentamicin resistance, respectively.*

### Construction of Deletion Mutants

To create the in-frame deletion of *mqsR, mqsA*, and *mqsRA* in *P. putida* KT2440, 5′ and 3′ homologous arm segments of 0.9–1.5 kb were generated by PCR from genomic DNA, and each amplicon was then ligated with Gm^R^-encoding cassette (from pPS856) into pEX18Ap to produce the replacement plasmids as described previously (Hoang et al., 1998). For example, to delete *mqsA*, 1334 bp upstream and 962 bp downstream fragments of *mqsA* ORF were generated by PCR (primers used were listed in Supplementary Table S1) using *P. putida* KT2440 genomic DNA. PCR product of each amplicon was then ligated with Gm^R^-encoding cassette into pEX18Ap which contains the *sacB*-based counter-selection marker to create the replacement plasmid. Next, the replacement plasmid was transformed into KT2440 by electroporation and the resulting deletion mutants (*mqsA* gene was replaced by Gm via homologous recombination) were selected on LB plate supplemented with gentamicin (Hoang et al., 1998). Deletion of the *mqsR* gene and the *mqsRA* operon were performed following similar steps, and all three deletion mutants were confirmed by PCR and DNA sequencing with primers listed in Supplementary Table S1.

### Construction of Plasmids

To overexpress *mqsR, mqsA*, and *mqsRA* in *E. coli* and *P. putida* KT2440 hosts, the full coding region of *mqsR, mqsA*, and *mqsRA* were amplified with primer pairs listed in Supplementary Table S1 using *P. putida* KT2440 genomic DNA as the template. PCR products were purified using a gel extraction kit (Qiagen, Hilden, Germany), digested with *Eco*RI (or *Nhe*I for pHGE-base plasmids) and *Hin*dIII, and were purified with a PCR product purification kit (Qiagen). The purified PCR products were ligated into the pMQ70 and pHGE expression plasmids and transferred into *E. coli* WM3064 and *P. putida* KT2440, respectively. The correct constructs were verified by DNA sequencing using primer pairs pMQ70-f/r and pHGE-f/r.

For purification of the MqsA protein and MqsR/MqsA complex, the coding region of *mqsA* and *mqsRA* were amplified with primer pairs pET28b-*mqsA*-f/-r to make pET28b-*mqsA*-His, pET28b-*mqsRA*-f/pET28b-*mqsRA*-His-r to make pET28b-*mqsRA*-CHis (Supplementary Table S1) using *P. putida* KT2440 genomic DNA as the template, PCR products were purified using a gel extraction kit (Qiagen), and digested with *Nco*I and *Hin*dIII, and were purified with a PCR product purification kit (Qiagen). The purified PCR products were ligated into the pET28b plasmid and transferred into *E. coli* BL21. The correct constructs were verified by DNA sequencing using primer T7.

### Construction of Reporter Strains for Promoter Activity Assays

For the promoter activity assay, DNA fragments containing 409 nt upstream of the translational start site of *mqsR*, a 328 nt upstream of the translational start site of *PP_3288*, and a 357 nt upstream of the translational start site of *algU* were selected as the promoter regions. pHGR01-P*mqsRA-lacZ* contains the promoter region of *mqsRA*, pHGR01-P*mqsRA-mqsA-lacZ* contains the promoter region and the ORF of the *mqsA.* Both of the fragments were digested with *Eco*RI and *Hin*dIII and cloned into the promoter-less *lacZ*-fusion vector pHGR01 digested with the same two enzymes. Constructed pHGR01-P*mqsRA-lacZ* was verified by DNA sequencing using the primer pair pHGR01-f/-r listed in Supplementary Table S1. pHGR01-P*PP_3288-lacZ* and pHGR01-P*algU-lacZ* were constructed following the same procedure. The resulting plasmids were verified by DNA sequencing and transformed into *E. coli* WM3064 strain. Mid-log phase (OD_600_ ∼ 0.7) cells of the indicated strains carrying the reporter plasmids were collected by centrifugation and washed with phosphate buffered saline. The cell soluble protein and β-galactosidase activity were determined using previously described protocols (Wu et al., 2011).

### Protein Expression and Purification

MqsA or the MqsR/MqsA complex with a hexahistidine tag at the C-terminus of MqsA and the MqsR/MqsA complex without any tag were purified using *E. coli* BL21 with pET28b-*mqsA-*His, pET28b-*mqsRA-*CHis, and pET28b-*mqsRA*. Strains were grown in LB with kanamycin (50 μg/ml) and were induced with 1 mM IPTG at a turbidity of 0.1 at 600 nm for 5 h. Cells were collected and resuspended in lysis buffer [50 mM potassium phosphate buffer (pH 8.0), 300 mM NaCl, and protease inhibitor cocktail (Sigma–Aldrich, USA)]. Samples were sonicated using a Sonic Dismembrator (Ningbo dongzhi, China) at level 2 for 5 min on ice. Ni-NTA resin (Qiagen) was used according to the manufacturer’s protocol. Purified proteins were desalted by passage on disposable Sephadex G-25 pre-packed PD-10 columns pre-equilibrated in 20 mM Tris-HCl buffer (pH 8.0), and the protein concentration was measured by the Bi Yuntian BCA assay kit (Haimen, Jiangsu, China). Tricine–SDS–PAGE was performed as previously described (Baba et al., 2006). A total of 20 μg of protein from each sample was loaded for SDS–PAGE.

### Quantitative Real-Time Reverse-Transcription PCR (qRT-PCR)

Total RNA was isolated as described previously (Ren et al., 2004). The cDNA synthesis was conducted using reverse transcription (Promega, Madison, WI, USA). Total cDNA (50 ng) was used for qRT-PCR using the Step One Real-Time PCR System (Applied Biosystems StepOne Real-Time PCR System, USA). Primers used for qRT-PCR are listed in Supplementary Table S1, and the level of *16S rRNA* gene transcript was used to normalize the gene expression data, and fold change of each gene was calculated as described previously (Pfaffl, 2001).

### Attached Biofilm Assay

Attached biofilm formation was assayed in 96-well polystyrene plates with 0.1% crystal violet staining as described previously (Pratt and Kolter, 1998). To remove growth effects, biofilm formation was normalized by dividing the maximal bacterial growth (measured by the turbidity at 620 nm) for each strain. The assay was repeated twice with at least three independent cultures.

### Electrophoretic Mobility Shift Assay (EMSA)

Electrophoretic mobility shift assays (EMSAs) were performed as previously described (Kim et al., 2010). The DNA probes were amplified by PCR using primers listed in Supplementary Table S1. The PCR products were gel purified and labeled with biotin using the Biotin 3′-end DNA Labeling Kit (Pierce, Rockford, IL, USA). For the binding reactions, biotin-probes (0.05 pmol DNA) were incubated with purified MqsA-His protein for 1 h at room temperature. The DNA binding reactions were performed with the non-specific competitor DNA (poly dI-dC) and NP-40 in buffer containing 10 mM HEPES (pH 7.3), 20 mM KCl, 1 mM MgCl_2_, and 5% glycerol in a total volume of 20 μl at 25°C for 1 h. Aliquots of binding reaction mixtures were then electrophoresed on 6% DNA acrylamide retardation gels (Invitrogen, Carlsbad, CA, USA) at 100 V in 0.5 × TBE buffer (10 mM Tris borate at pH 8.3 and 2 mM EDTA) for 90 min. DNA was transferred to a nylon membrane at 390 mA for 40 min, followed by UV crosslinking at 302 nm for 20 min. Chemiluminescence was performed with the LightShift Chemiluminescent EMSA Kit (Thermo Scientific, Rockford, IL, USA) according to the manufacturer’s protocol.

### Persistence Assay

Persistence of *P. putida* KT2440 was measured by time-dependent killing experiments (Dorr et al., 2010). To test the formation of persister cells in *P. putida* KT2440, overnight bacterial culture was re-inoculated into fresh LB medium (at a turbidity of 0.1 at 600 nm) and grown to an OD_600_ of 1.0. For strains carrying pHGE-based plasmids, overnight cultures were diluted to a turbidity of 0.1 and grown to a turbidity of 0.5, then 1 mM IPTG was used to induce gene expression for 2 h, and the turbidity was adjusted to 1.0. Then the bacterial cultures were exposed to 1.25 μg/ml ciprofloxacin. Cell viability (CFU/ml) was determined at the time points indicated. Cell survival was determined by drop assay.

### Phylogenetic Analysis

MqsR homologs were retrieved from ortholog clusters (K13651) in the KEGG ORTHOLOGY database and the data in a previous constructed phylogenetic tree of MqsR (Makarova et al., 2009). By excluding a few nearly identical sequences from different strains of the same bacterial species, the data set of total 133 genes were used for our phylogenetic analysis. The amino acid sequences were aligned with the MAFFT program (L-INS-i) (Katoh and Standley, 2013) and the poorly aligned sites were deleted by trimAl (with gt 0.8) (Capella-Gutierrez et al., 2009). A final data set of 98 amino acid sites was used for the maximum likelihood (ML) phylogenetic analysis by the W-IQ-TREE (Trifinopoulos et al., 2016). The best-fit substitution model was automatically determined and the reliability of internal branches was tested by 1000 ultrafast bootstrap replicates (Minh et al., 2013) in the W-IQ-TREE web interface. The ML tree was rooted to split the two groups of MqsR described previously (Makarova et al., 2009) and further annotated by the iTOL tool (Letunic and Bork, 2016).

### Palindrome Search

The motifs 5′-ACCT (N)_3_ AGGT-3′ were identified using the Biostrings and BSgenome libraries in the R statistical package (version 2.9.2) and Fuzznuc (EMBOSS) (Rice et al., 2000).

### Statistical Analysis

Data are presented as means ± SE of three or more independent cultures. Statistical significance was assessed using two-tailed unpaired Student’s *t*-test.

## Results

### Phylogenetic Analyses of MqsR/MqsA Homologs

**FIGURE 1:**
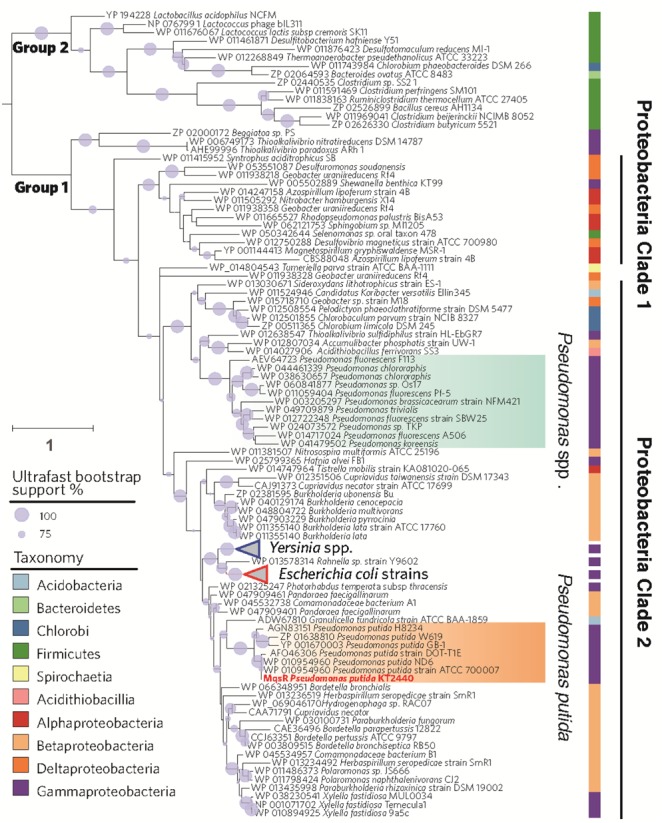
**Phylogenetic tree of MqsR homologs.** The maximum likelihood tree was rooted to split into two groups of MqsR homologs in 133 bacterial genomes. The branch of MqsR of *Pseudomonas putida* KT2440 is highlighted in red. Branch lengths are proportional to the number of amino acid substitutions (see scale bar in left) and gray circle on branches show ultrafast bootstrap support values that are greater than 50%. Taxa are colored according to the taxonomy.

A total of 143 genes encoding for MqsR homologs were found in various bacterial genomes by searching the KEGG ORTHOLOGY database. As shown in **Figure 1**, MqsR toxin seems to be divergent in different genera. In consistent with what has been reported earlier (Makarova et al., 2009), MqsR toxin belongs to two phylogenetically distinct groups (**Figure 1**). Group 1 comprises mostly proteobacterial MqsR toxins, while Group 2 comprises MqsR toxins of Firmicutes, Bacteroidetes, and Chlorobi. MqsR homologs of Proteobacteria basically fall into two separate clades. The Proteobacteria clade 1 mainly comprises MqsRs of α-Proteobacteria and δ-Proteobacteria, and the Proteobacteria clade 2 comprises those of β-Proteobacteria and γ-Proteobacteria. MqsR homologs of *P. putida* seem more closely related with that of *E. coli* and *Yersinia* spp. than other *Pseudomonas* MqsR toxins.

### Identification of MqsR/MqsA as a TA Pair in *P. putida* KT2440

**FIGURE 2:**
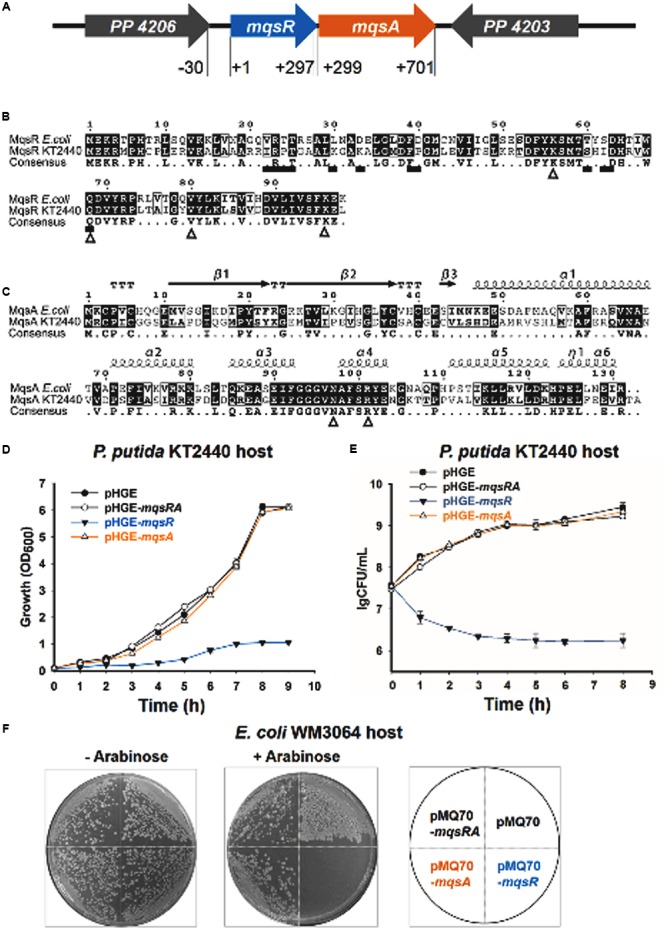
**MqsR (PP_4205) is toxic and MqsA (PP_4204) neutralizes the toxicity of MqsR. (A)** The *mqsRA* locus in *P. putida* KT2440. The number indicates the relative position to the start codon of *mqsR*. **(B)** Comparison of amino acid sequence of MqsR in *Escherichia coli* K-12 and in *P. putida* KT2440. Triangles below the consensus sequences (K59, Q69, V81, and K96) show the important residues for toxicity of *E. coli* MqsR and squares show the MqsA-interacting residues (Brown et al., 2009). **(C)** Comparison of amino acid sequences and predicted secondary structures of MqsA in *E. coli* K-12 and in *P. putida* KT2440. Triangles below the consensus sequences (N97 and R101) show the important residues for MqsA to bind DNA and for transcription regulation in *E. coli* (Brown et al., 2009). **(D)** Growth of the *P. putida* KT2440 strains harboring the pHGE-based plasmids were induced with 0.5 mM IPTG at OD_600_∼0.1. **(E)** Cell viability (CFUs/ml) was determined at the time points indicated. Error bars indicate standard error of mean (*n* = 3) in **D,E**. **(F)** *E. coli* WM3064 hosts harboring pMQ70-based plasmids were streaked on LB plates supplemented with 100 μg/ml ampicillin with or without 10 mM L-arabinose.

Putative type II TA systems in the *P. putida* KT2440 genome were predicted with the web-based tool RASTA-Bacteria (Sevin and Barloy-Hubler, 2007). Two neighboring genes, *PP_4205* and *PP_4204*, encoding two proteins of 98 aa and 133 aa, were identified as a putative TA pair (**Figure 2A** and Supplementary Figure S1). PP_4205 shares 58 % identity with MqsR in *E. coli*, and the four amino acid residues (Lys^56^, Gln^69^, Tyr^81^, and Lys^96^) which are important for MqsR toxicity of *E. coli* (Brown et al., 2009) are also conserved in *P. putida* KT2440 (**Figure 2B**). PP_4204 shares 45% identity with MqsA in *E. coli* at the amino acid sequence level, the two residues Asn^97^ and Arg^101^ that are important for MqsA to bind DNA and for transcription regulation in *E. coli* (Brown et al., 2009) are also conserved in *P. putida* KT2440 (**Figure 2C**).

To explore the function of this TA pair in *P. putida* KT2440, we first tested the toxicity of the two-gene cassette. The coding regions of the two genes were cloned into the pHGE plasmid to construct pHGE-*PP_4204* (pHGE-*mqsA*) and pHGE-*PP_4205* (pHGE-*mqsR*) using genomic DNA of *P. putida* KT2440 as template. When transformed into *P*. *putida* KT2440, cells expressing MqsR using pHGE-*mqsR* exhibited a notable decrease in cell growth and viability as shown by the reduction in turbidity at 600 nm (OD_600_) and colony forming units (CFUs) (**Figures 2D,E**). In contrast, expression of MqsA using pHGE-*mqsA* did not affect cell growth in *P. putida* KT2440 (**Figures 2D,E**).

To further determine whether the neighboring protein MqsA can neutralize the toxicity of MqsR, we constructed the pHGE-*mqsRA* plasmid to co-express MqsA and MqsR in *P. putida* KT2440 cells. As expected, MqsA completely neutralized the toxicity of MqsR (**Figures 2D,E**). Moreover, co-expression of MqsA with MqsR effectively inhibited cell death caused by MqsR overproduction (**Figures 2D,E**). To further test the toxicity of these two proteins in a different host, we constructed and transferred pMQ70-based constructs into *E. coli* WM3064 host. As expected, MqsR was toxic while MqsA neutralized the toxicity of MqsR in *E. coli* host (**Figure 2F**). Therefore, PP_4205 and PP_4204 in *P. putida* KT2440 consist of a type II TA pair.

### *mqsR* and *mqsA* Are Co-transcribed

To determine whether *mqsR* and *mqsA* are co-transcribed, we designed a forward primer in the first gene *mqsR* (*mqsR-*RT-f) and a reverse primer in the second gene *mqsA* (*mqsA-*RT*-*r) (Supplementary Figure S1) and conducted a reverse transcription polymerase chain reaction (RT-PCR) assay. As shown in Supplementary Figure S2, a single band of 707 bp was amplified using cDNA synthesized from total RNA as the template, and the PCR product was sequenced to be the region among the two primers, indicating *mqsR* and *mqsA* form a single operon (Supplementary Figure S2, lane 3). As controls, the same band was detected using genomic DNA (Supplementary Figure S2, lane 2) as the template but not for total RNA (Supplementary Figure S2, lane 4). This result was also supported by qRT-PCR results as the same levels of transcripts were determined for *mqsR* and *mqsA* gene in the normal growing *P. putida* KT2440 cells (Supplementary Table S2). Collectively, these results demonstrate that *mqsR* and *mqsA* are co-transcribed.

### MqsR Interacts with MqsA *In Vivo*

**FIGURE 3:**
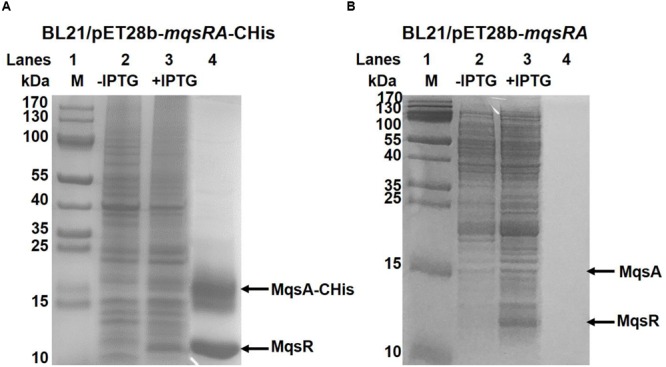
**MqsR and MqsA form a complex *in vivo*. (A)** His-tagged MqsA and untagged MqsR were produced from pET28b-*mqsRA-*CHis in *E. coli* BL21. MqsA-CHis (15.5 kDa) and MqsR (11.1 kDa) were induced with IPTG (lane 3). MqsR was co-purified with MqsA-CHis (lane 4). Cells that were not induced were used as the negative control (lane 2). **(B)** Cells harboring pET28b-*mqsRA* was used as negative control. Neither untagged MqsA nor untagged MqsR bound to the Ni-NTA agarose beads (lane 4). Protein marker (M) was loaded in lane 1 in **A,B**.

Direct protein-protein interaction between toxin and antitoxin *in vivo* is a typical feature of type II TA systems (Brown et al., 2011). To determine whether MqsR binds to MqsA, we performed a pull-down assay using pET28b-*mqsRA*-CHis to co-express a C-terminus hexahistidine tagged (His-tagged) MqsA along with an untagged toxin MqsR (Yao et al., 2015; Guo et al., 2016). Affinity purification using Ni-NTA agarose beads and subsequent Tricine–SDS–PAGE revealed that another small protein was pulled down in addition to His-tagged MqsA (15.51 kDa) (**Figure 3A**, lane 4), and this small protein was verified to be MqsR (11.13 kDa) toxin by mass spectrometry (Supplementary Table S3). We also constructed pET28b-*mqsRA* to express untagged MqsR and untagged MqsA, and neither of them could bind to Ni-NTA beads (**Figure 3B**, lane 4), suggesting MqsR was purified due to its interaction with MqsA-His. Hence, we showed that inhibition of MqsR toxicity by antitoxin MqsA was most likely due to the direct interaction between the two proteins.

### MqsA Represses Transcription of the *mqsRA* Operon

**FIGURE 4:**
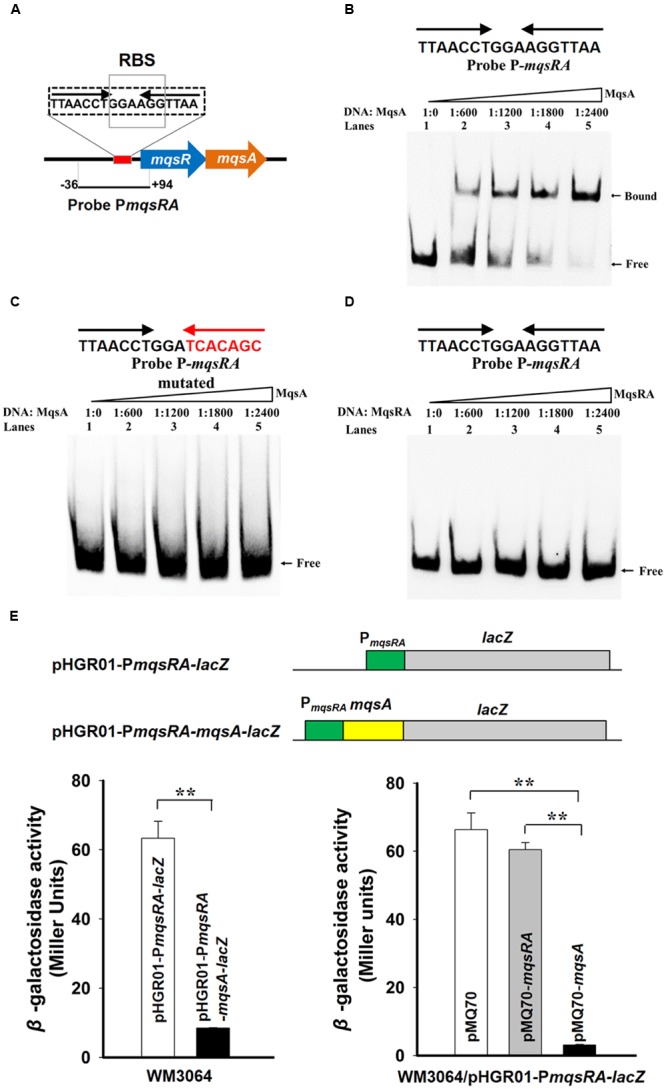
**MqsA binds the *mqsRA* operon. (A)** The promoter region of the *mqsRA* operon contains a 17 bp palindrome 5′-TTAACCT GGA AGGTTAA-3′ and the ribosome-binding site (RBS) for *mqsR* which is marked with boxes. **(B)** Electrophoretic mobility shift assay (EMSA) results showed that MqsA-CHis bound and shifted the promoter region of *mqsRA* (P*mqsRA*). **(C)** The binding of MqsA to P*mqsRA* was abolished after random mutating the palindrome to 5′-TTAACCT GGA TCACAGC-3′. **(D)** MqsR/MqsA complex did not bind to P*mqsRA*. DNA probe P*mqsRA* used for EMSAs is the same as **A**. **(E)** Schematic diagram of the two constructed reporter plasmids for promoter activity assay. Mid-log phase WM3064 cells carrying two different reporter plasmids (lower left) and overexpressing *mqsA* or *mqsRA* in the presence of one of the reporter plasmids (lower right) were tested for β-galactosidase activity. Three independent cultures for each strain were used and the data are shown as means ± standard deviations. Asterisks represent a statistically significant difference in **E** (*P* < 0.01 was shown in ^∗^ and *P* < 0.001 was shown in ^∗∗^; *n* = 3).

It has been shown that the type II antitoxin alone or the TA complex binds to its promoter and negatively regulates the transcription of the TA operon (Yamaguchi et al., 2011). Analysis of the promoter region of *mqsRA* operon revealed that it contains a 17 bp palindrome 5′-TTAACCT GGA AGGTTAA-3′, and the palindrome is overlapped the ribosome-binding site (RBS) for *mqsR* (**Figure 4A**). To check whether MqsA binds to the promoter region, we first performed EMSA using purified MqsA protein (Supplementary Figure S3) and a PCR product of 130 bp that includes the promoter region of the TA operon (**Figure 4A** and Supplementary Figure S1). MqsA specifically bound to the *mqsRA* promoter region in a concentration-dependent manner (**Figure 4B**). To determine whether MqsA binds to the palindromic sequence, we performed EMSA using a mutant DNA probe with the palindrome disrupted, and results showed that MqsA did not bind to the mutant DNA probe (**Figure 4C**). When the MqsR/MqsA complex purified using pET28b-*mqsRA*-CHis was used for EMSA, the TA complex could not bind and shift the *mqsRA* promoter (**Figure 4D**). This result is consistent with the previous work in *E. coli* which demonstrated that the MqsR/MqsA complex was unable to bind DNA when MqsR and MqsA are present at 1:1 ratio (Brown et al., 2013). These *in vitro* results suggest that the autoregulation of the *mqsRA* operon relies on the ratio of the toxin protein to the antitoxin protein present in the cell. Thus, similar to MqsR in *E. coli*, MqsR in *P. putida* KT2440 does not function as a transcriptional co-repressor, but blocks the antitoxin MqsA to bind DNA when they are present at approximately 1:1 ratio in the cell.

Moreover, a promoter activity assay using a transcriptional fusion was employed to study the autoregulation of the MqsR/MqsA TA pair *in vivo*. We cloned the promoter region of *mqsRA* into the pHGR01 plasmid to create the pHGR01-P*mqsRA-lacZ* reporter plasmid. To examine the effect of MqsA, we also fused the promoter region of *mqsRA* and the coding region of *mqsA* to the same promoterless *lacZ* reporter gene to create pHGR01-P*mqsRA-mqsA-lacZ* (**Figure 4E**, up panel). The promoter activity was decreased from 63 ± 4.0 miller units (MU) in the *E. coli* WM3064 cells carrying the pHGR01-P*mqsRA-lacZ* plasmid to 8 ± 0.1 MU in cells harboring the pHGR01-P*mqsRA-mqsA-lacZ* (**Figure 4E**, left panel), suggesting that the presence of MqsA repressed the promoter activity of pHGR01-P*mqsRA-lacZ*. In addition, we also overexpressed *mqsA* using pMQ70-*mqsA* in the reporter strain WM3064/pHGR01-P*mqsRA-lacZ*. The promoter activity reduced from 66.0 ± 5.0 MU to 3.0 ± 0.2 MU after overexpressing MqsA in the *E. coli* WM3064 cells. However, when TA complex was overproduced by pMQ70-*mqsRA*, the promoter activity was not significantly reduced in the reporter strain (**Figure 4E**, right panel). Therefore, MqsA represses transcription of the *mqsRA* operon, and the presence of MqsR blocks the activity of MqsA to bind DNA when it is co-expressed.

### MqsA and MqsR Affect Biofilm Formation and Persistence

**FIGURE 5:**
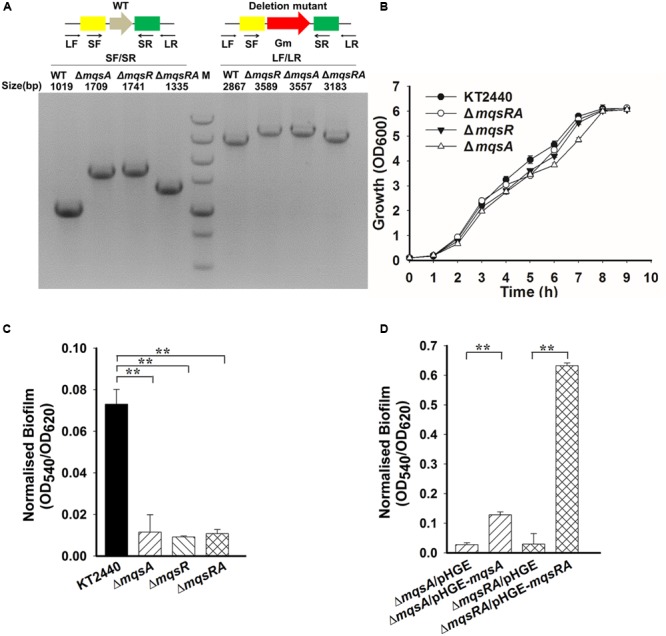
**MqsA and MqsR affect biofilm formation. (A)** Deletion of *mqsR, mqsA*, and *mqsRA* operon in *P. putida* KT2440 are checked by PCR. PCR products are indicated with the expected sizes using genomic DNA from the wild-type (WT) and the deletion mutants. M indicates DNA ladder (500, 750, 1000, 1500, 2000, 3000, and 5000 bp). **(B)** Growth of different strains in LB medium. **(C)** Attached biofilm formation of different strains quantified in the 96-well polystyrene plate. **(D)** Attached biofilm formation of different strains (with 0.5 mM IPTG) using the 96-well polystyrene plate assay in LB medium. Three independent cultures for each strain were used and the data are shown as means ± standard deviations. Asterisks represent a statistically significant difference in **C** and **D**. (*P* < 0.01 was shown in ^∗^ and *P* < 0.001 was shown in ^∗∗^; *n* = 3).

To probe the physiological function of MqsR/MqsA in *P. putida* KT2440, we constructed in-frame deletion mutants of *mqsR, mqsA*, and *mqsRA* in *P. putida* KT2440. The deletion mutants were verified by PCR (**Figure 5A**) followed by DNA sequencing to make sure no other mutations occurred in the neighboring gene. Deletion of the toxin gene or the antitoxin gene did not significantly affect cell growth (**Figure 5B**). Since TA systems are related to biofilm formation (Wang and Wood, 2011), attached biofilm formation was tested for the deletion mutants using the 96-well polystyrene plate assay. Attached biofilm formation was reduced 8.0 ± 0.1-fold, 6.3 ± 0.1-fold, and 6.8 ± 0.1-fold after 2 h for the *mqsR, mqsA*, and *mqsRA* deletion mutants compared to the wild-type KT2440 strain, respectively (**Figure 5C**). Moreover, overexpressing *mqsA* via pHGE-*mqsA* in the *mqsA* deletion mutant strain increased biofilm formation and overexpressing *mqsRA* via pHGE-*mqsRA* also increased biofilm formation (**Figure 5D**). We did not perform complementation study for *mqsR* due to the high toxicity of MqsR when overexpressed in *P. putida* KT2440.

**FIGURE 6:**
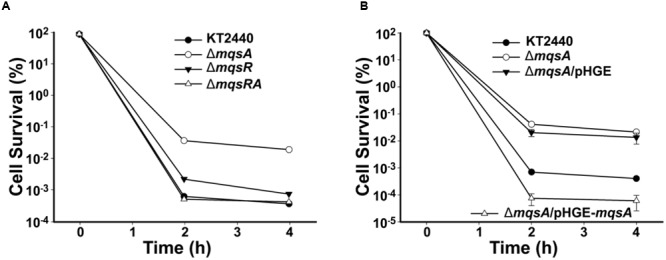
**Role of MqsR and MqsA in persister cell formation. (A)** Survival for the *P. putida* KT2440 wild-type, Δ*mqsR*, Δ*mqsA*, and Δ*mqsRA* strains after challenging with 1.25 μg/ml ciprofloxacin (added at OD_600_∼1.0 at *T* = 0). **(B)** Survival for the *P. putida* KT2440 wild-type, Δ*mqsA*, Δ*mqsA*/pHGE, and Δ*mqsA*/pHGE-*mqsA* strains after challenging with 1.25 μg/ml ciprofloxacin at OD_600_∼1.0 (0.5 mM IPTG was added at *T* = 0). At indicated time points, survival rates were determined and error bars indicate standard error of mean (*n* = 3) in **A,B**.

To test the role of MqsR/MqsA in persistence, persister cell assay was performed for the *mqsA* or *mqsR* mutant strains using a high dose of ciprofloxacin. As shown in **Figure 6A**, deletion of *mqsA* increased the formation of persister cells 52.7 ± 4.2-fold, while deletion of *mqsR* or *mqsRA* did not significantly affected persister cell formation. We further complemented MqsA in the Δ*mqsA* strain via pHGE-*mqsA*, and results demonstrated that overproduction of MqsA inhibited the formation of persister cells 219.7 ± 10.2-fold compared to the Δ*mqsA*/pHGE strain (**Figure 6B**). Since the deletion of *mqsA* would activate the expression of *mqsR*, we conducted qRT-PCR to check the expression level of *mqsR* in the *mqsA* deletion mutant strain. As shown in Supplementary Table S2, the transcription of the toxin gene *mqsR* was induced 2.0 ± 0.1-fold in the Δ*mqsA* strain compared to the wild-type strain. In addition, expression of *mqsR* was repressed 5.6 ± 0.2-fold in the Δ*mqsA*/pHGE-*mqsA* strain compared to the Δ*mqsA*/pHGE-*mqsA* strain. Since there was no difference in persister cell formation between the Δ*mqsR* and Δ*mqsRA* strain, and deletion of *mqsR* did not affect *mqsA* expression as shown in the Δ*mqsR* strain (Supplementary Table S2), MqsR should be mainly responsible for the increased persistence detected in the *mqsA* deletion mutant and also responsible for the reduced persistence in the *mqsA* complementation strain in *P. putida* KT2440.

### MqsA Negatively Regulates Other Genes

We have previously demonstrated that MqsA in *E. coli* can bind to the promoter of *rpoS* and *csgD* via the MqsA-like palindrome 5′-ACCT (N)_3_ AGGT-3′ where N is any nucleotide. We found that although MqsR and MqsA in *E. coli* K-12 and in *P. putida* KT2440 share medium level of identify at the amino acid sequence level, the palindrome where MqsA binds are the same in these two strains. However, different from *E. coli* K-12, there is no MqsA-like palindrome in the gene encoding the stationary sigma factor RpoS in *P. putida* KT2440, and moreover, the master regulator CsgD for curli and cellulose production is not found in *P. putida* KT2440. Therefore, we conducted a whole-genome search to identify intergenic regions with 5′-ACCT (N)_3_ AGGT-3′ in *P. putida* KT2440. As shown in **Table 2**, the MqsA-like palindrome was found in the upstream region of another three genes in *P. putida* KT2440, including *algU, PP_3288*, and *nadB*.

**Table 2:** List of all genes with MqsA-like palindrome 5′-**ACCT** (N)_3_ **AGGT**-3′ in the promoter region in *Pseudomonas putida* KT2440 identified by the whole-genome search.

Gene ID	Gene name	Gene description	MqsA-like palindrome (5′-3′)
PP_4205	*mqsR*	Toxin of the toxin/antitoxin pair	**ACCT** GGA **AGGT**
PP_1427	*algU* (*rpoE* or σ^22^)	RNA polymerase sigma factor	**ACCT** GCC **AGGT**
PP_3288	*PP_3288*	Universal stress protein	**ACCT** CAG **AGGT**
PP_1426	*nadB*	L-aspartate oxidase	**ACCT** GGC **AGGT**

*The bold letters show the palindrome sequence.*

**FIGURE 7:**
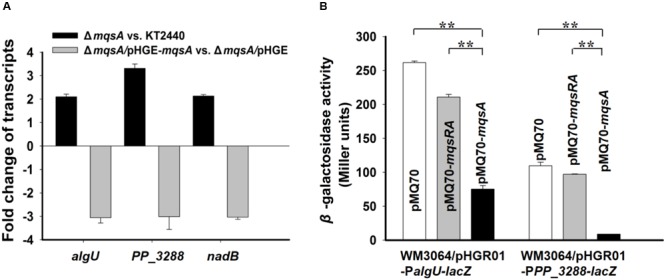
**MqsA negatively regulates *algU, PP_3288* and *nadB*. (A)** Fold changes of expression of three genes in *ΔmqsA* vs. KT2440, and *ΔmqsA/*pHGE-*mqsA* vs. *ΔmqsA/*pHGE were quantified by qRT-PCR. **(B)** Mid-log phase cells of the reporter strains harboring pMQ70, pMQ70-*mqsRA*, and pMQ70-*mqsA* (with 10 mM L-arabinose) were tested for β-galactosidase activity, respectively. Three independent cultures for each strain were used and the data are shown as means ± standard deviations. Asterisks represent a statistically significant difference. (*P* < 0.01 was shown in ^∗^ and *P* < 0.001 was shown in ^∗∗^; *n* = 3).

AlgU is one of the ECF sigma factors in *Pseudomonas*, which is required for full resistance of *P. aeruginosa* to oxidative stress and responsible for alginate biosynthesis (Martinezsalazar et al., 1996; Tart et al., 2005; Bazire et al., 2010). PP_3288 is predicted to be a universal stress family protein but its exact function has not been elucidated. NadB is L-aspartate amino acid oxidase and its activity is required for nicotinamide adenine dinucleotide (NAD) biosynthesis in both aerobic and anaerobic conditions in *E. coli* (Seifert et al., 1990). We first conducted qRT-PCR to check the expression of these three genes in the *mqsA* deletion mutant strain as compared to the wild-type *P. putida* KT2440 strain, and the transcription of the three genes were significantly induced (about twofold to threefold) as compared to the wild-type strain. In addition, overexpressing *mqsA* via pHGE-*mqsA* repressed the expression of these genes about threefold (**Figure 7A**). Next, we performed a promoter activity assay using a transcriptional fusion to study the regulation of the MqsA with *algU in vivo*. We cloned the promoter region of *algU* into the pHGR01 plasmid to create the reporter plasmid pHGR01-P*algU-lacZ.* The promoter activity was decreased from 216.5 ± 2.3 MU to 75.2 ± 5.2 MU after overexpressing *mqsA* via pMQ70-*mqsA* in the *E. coli* WM3064 that harbored the reporter plasmid (**Figure 7B**). However, when the TA complex MqsR/MqsA was overproduced via pMQ70-*mqsRA*, the promoter activity was not significantly changed. Similar results were obtained for the *PP_3288* gene. The promoter activity was decreased from 109.6 ± 5.1 MU to 8.8 ± 0.1 MU after overexpressing *mqsA* in the *E. coli* WM3064 that harbored the pHGR01-P*PP_3288-lacZ* plasmid, and when the MqsR/MqsA complex was overproduced by pMQ70-*mqsRA*, the promoter activity was not significantly reduced (**Figure 7B**). Collectively, these results suggest that MqsA regulates more than its own locus in *P. putida* KT2440, and this activity is also blocked by MqsR when it is co-expressed.

## Discussion

In this study, we present evidence to support that MqsR (PP_4205) and MqsA (PP_4204) in *P. putida* KT2440 form a type II TA system. These results are: (i) *mqsR* and *mqsA* form an operon (*PP_4205*-*PP_4204*), and they are co-transcribed; (ii) both of them encode small proteins; (iii) MqsR functions as a toxin that inhibits growth, and MqsA blocks the toxicity of MqsR by direct protein–protein interaction; (iv) the antitoxin MqsA regulates transcription of the *mqsRA* operon by binding to its promoter; and (v) the binding of MqsR to MqsA inhibits its DNA binding ability. These features fit well with the MqsR/MqsA pair in *E. coli* K-12. Although these homologous proteins share medium level of amino acid identity, the previously identified key residues for MqsR toxicity and for the DNA binding of MqsA in *E. coli* K-12 are also conserved in *P. putida* KT2440 (**Figures 2B,C**). MqsR homologs from diverse bacterial lineages belong to two groups (**Figure 1**). In our analysis, Group 1 basically falls into two separate clades comprising different classes of the Proteobacteria phylum. In particular, MqsR homologs of *Pseudomonas* spp. and different *P. putida* strains further nest into two different clades (**Figure 1**). Thus, further study is needed to explore the function of MqsR in different phylogenetic groups and in different clades.

The MqsR/MqsA system has been shown to regulate biofilm formation, curli/cellulose production, stress resistance, motility, and persistence in *E. coli* (Barrios et al., 2006; Kim et al., 2010; Wang et al., 2011; Hong et al., 2012). Deletion of *mqsR* reduced biofilm formation suggests that MqsR increases biofilm formation in *P. putida* KT2440. Furthermore, MqsR increases persister cell formation in *P. putida* KT2440. The MqsR toxin identified in *E. coli* belongs to the RelE superfamily and functions as ribosome-independent mRNase (Yamaguchi et al., 2009; Christensen-Dalsgaard et al., 2010). MqsR cleaves mRNAs *in vivo* and *in vitro*, the endoribonuclease activity of the MqsR was inhibited by MqsA (Yamaguchi et al., 2009). Primer extension analysis of the *in vivo* and *in vitro* cleaved RNA indicated that MqsR cleaves RNA before or after the G residue in GCU sequences, and cleavage of some GCA/G/C sites could also be detected *in vivo* (Yamaguchi et al., 2009; Christensen-Dalsgaard et al., 2010). Our attempt to purify the MqsR was unsuccessful due to its high toxicity, similar to previous studies (Robson et al., 2009). Co-purification of MqsR and MqsA resulted in strong interaction between MqsR and MqsA, which made it difficult to separate them without affecting the activity of the toxin. It has been reported that overproduction of MqsR caused elongated cells in *E. coli* and in *X. fastidiosa* (Yamaguchi et al., 2009; Merfa et al., 2016). However, overexpressing MqsR in *P. putida* KT2440 did not show any alteration in cell morphology (Supplementary Figure S4). Moreover, the type I toxin gene *ralR* and the type V toxin gene *ghoT* which are enriched when MqsR is produced in *E. coli* (Wang et al., 2013; Guo et al., 2014) are both absent in *P. putida* KT2440. These results suggest that MqsR should have different cellular targets and is involved in specific biological processes in different bacteria.

Except for binding to its cognate toxin MqsR and to its own promoter, antitoxin MqsA also regulates other important loci. Specially, we have demonstrated that the decrease of biofilm in *E. coli* is due to MqsA binding to the promoter of *rpoS* and repressing expression of *rpoS*, decreased levels of RpoS reduces the concentration of c-di-GMP (Wang et al., 2011). The binding of MqsA to the promoter of *cgsD* is also reported which confirms the role of MqsA in regulating other genetic loci (Soo and Wood, 2013). The binding of MqsA to the promoter region of *rpoS* and *csgD* are through the MqsA-like palindrome 5′-ACCT (N)_3_ AGGT-3′ present in the promoters of these two genes. Moreover, the sequences of the three nucleotides spacer do not seem to have any effect on MqsA binding affinity to DNA (Brown et al., 2011). In this study, we found that, although there is very low sequence similarity at the promoter region of the *mqsRA* operon in *P. putida* KT2440 and in *E. coli* K12, the palindromes where MqsA binds are the same in these two strains. In *E. coli*, the removal of *mqsR* increased biofilm formation (Kim et al., 2010), while the removal of the *mqsRA* operon reduced biofilm formation (Kasari et al., 2010). However, in *P. putida* KT2440, deletion of *mqsR* and deletion of *mqsA* both reduced biofilm formation, and the role of MqsR and MqsA in biofilm formation was further shown by the complementation study in which overexpressing the TA complex or MqsA alone increased biofilm formation. Thus, it seems that both MqsR and MqsA are able to regulate biofilm formation of *P. putida* KT2440. Moreover, different from *E. coli* K-12 in which the removal of *mqsA* gene was lethal, the deletion of the antitoxin gene *mqsA* was not lethal in *P. putida* KT2440. The non-lethal effect of *mqsA* deletion in KT2440 could be partially explained by a low expression of the *mqsRA* operon and/or a relatively low level of MqsR production in the absence of its cognate antitoxin in the planktonic growth condition (Supplementary Table S2). A relatively lower expression of the *mqsRA* operon was also found in the planktonic growth condition in *E. coli* (Domka et al., 2007). Considering that there are two identical palindromes for the binding of MqsA in the promoter region of the *mqsRA* operon in *E. coli* but only one in *P. putida* KT2440, it is possible that the repression of MqsA on the *mqsR* transcription in *P. putida* KT2440 is less efficient than in *E. coli*. Deletion of the antitoxin gene *graA* of the GraT/GraA type II TA pair was also successful in *P. putida* KT2440; however, the level of *graT* mRNA was much higher in the *graA* deletion mutant than that of the wild-type strain (Tamman et al., 2014).

As a DNA binding protein, MqsA in *E. coli* binds to the promoter of *rpoS* gene and the *csgD* genes. However, different from *E. coli* K-12, there is no MqsA-like palindrome in the promoter region of the stationary sigma factor RpoS in *P. putida* KT2440. In addition, the master regulator CsgD for curli and cellulose production is not found in *P. putida* KT2440. Interestingly, we found another gene encoding an alternative sigma factor AlgU containing the MqsA-like palindrome in the upstream region in *P. putida* KT2440. ECF sigma factors are a group of alternative sigma factors whose target gene products often function outside the cytoplasm and many of them are related to virulence and pathogenesis in *Pseudomonas* (Lonetto et al., 1994). AlgU is a positive regulator of alginate production and negative regulator of flagella-associated motility in *P. aeruginosa* (Tart et al., 2005). Both alginate and flagella are important for the biofilm formation of *P. aeruginosa.* Alginate promotes cells to adhere to biotic and abiotic surfaces and can also function as the scaffolding material of *P. aeruginosa* biofilms (Halverson, 2009). Motility is necessary for the bacteria to reach surfaces suitable for biofilm formation and spreading of the biofilms (Otoole and Kolter, 1998; Klausen et al., 2003). RpoS and AlgU are both global regulators that control expression of many proteins in conjunction with various regulatory factors (Hengge, 2008; Schulz et al., 2015). Thus, both the toxin MqsR and antitoxin MqsA may play important regulatory roles in *P. putida* including biofilm formation and virulence. It remains interesting to investigate the regulation and impact of TA systems on the modulation of a single cell or in a population or during host–pathogen interaction.

## Author Contributions

XW conceived and designed the experiments, CS, YG, KT, ZW, BL, and ZZ performed the experiments and data analysis. XW and CS wrote the manuscript.

## Conflict of Interest Statement

The authors declare that the research was conducted in the absence of any commercial or financial relationships that could be construed as a potential conflict of interest.
