# Supplementary material for: MqsR/MqsA Toxin/Antitoxin System Regulates Persistence and Biofilm Formation in Pseudomonas putida KT2440
Source: Front Microbiol. 2017 May 9;8:840. doi: 10.3389/fmicb.2017.00840 (PMC5422877; doi:10.3389/fmicb.2017.00840)
Supplement: Supplementary file 1 [file Data_Sheet_1.PDF]

Supplementary Material

MqsR/MqsA Toxin/Antitoxin System Regulates Persistence and Biofilm Formation in *Pseudomonas putida* KT2440

Chenglong Sun[1,2], Yunxue Guo[1], Kaihao Tang[1], Zhongling Wen[1,2], Baiyuan Li[1], Zhenshun Zeng[1], Xiaoxue Wang[1*]

[1]Key Laboratory of Tropical Marine Bio-resources and Ecology, Guangdong Key Laboratory of Marine Materia Medica, RNAM Center for Marine Microbiology, South China Sea Institute of Oceanology, Chinese Academy of Sciences, Guangzhou, PR China

[2]University of Chinese Academy of Sciences, Beijing, PR China

*__Correspondence:__
Xiaoxue Wang
Email: xxwang@scsio.ac.cn
Tel: +86 20 89267515;
Fax: +86 20 89235490;

Keywords: Toxin/antitoxin system, MqsR/MqsA, biofilm, persistence, *Pseudomonas putida*.

Table. S1 Oligonucleotides used for plasmid construction, gene knockout, DNA sequencing and qRT-PCR. Restriction enzyme sites are underlined. f indicates forward primer and r indicates reverse primer.

**Purpose/Name**	**Sequence (5'-3')**
**Plasmid construction**	
pHGE-*mqsRA*-f	CCGGAATTCATGGCTACCAATGGTATGCGCC
pHGE-*mqsRA*-r	GCCCCCAAGCTTCTAGGCAGTACGCACTTCCTCAAAGAGT
pHGE-*mqsA*-f	CCGGAATTCATGAGATGTCCAATATGCGGC
pHGE-*mqsR*-r	GCCCCCAAGCTTCTACAACTCCTTGAAAGACACGATGAG
pMQ70-*mqsRA*-f	GCCCCCGCTAGCAAGAAGGAGATATACCC<br>GTGATGGAAAAGAGAATGCCTCAT
pMQ70-*mqsRA*-r	GCCCCCAAGCTTCTAGTGGTGGTGGTGGT<br>GGTGGGCAGTACGCACTTCCTCAAAGAGT
pMQ70-*mqsA*-f	GCCCCCGCTAGCAAGAAGGAGATATACCC<br>GTGATGAGATGTCCAATATGCGGC
pMQ70-*mqsR*-r	GCCCCCAAGCTTCTAGTGGTGGTGGTGGT<br>GGTGCAACTCCTTGAAAGACACGATGAG
pET28b-*mqsRA*-f	CTAGCCATGGGCATGGAAAAGAGAATGCCTCAT
pET28b-*mqsRA*-His-r	GCCCCCAAGCTTCTAGTGGTGGTGGTGGT<br>GGTGGGCAGTACGCACTTCCTCAAAGAGT
pET28b-*mqsRA*-r	GCCCCCAAGCTTCTAGGCAGTACGCACTTCCTCAAAGAGT
pET28b-*mqsA*-f	CTAGCCATGGGCATGAGATGTCCAATATGCGGC
pHGR01-P*mqsRA*-f	CCGGAATTCATGACCTGGCTGGCCTATG
pHGR01-P*mqsRA*-r	CGCGGATCCGTAATCATGGTCATGAGAAATTAACCTTCCAGGTT
pHGR01-P*PP_3288*-f	CCGGAATTCTCCCTGCTCAGTATTCTTCTCG
pHGR01-P*PP_3288*-f	CCCAAGCTTGTAATCATGGTCATGGCACACCTCTGAGGTTGCGA
pHGR01-P*algU*-f	CCGGAATTCCGTGTCGGGATGCCTTTA
pHGR01-P*algU*-r	CCCAAGCTTGTAATCATGGTCATGAACACTCCTCAGTGAACTCG
**PCR and DNA sequencing**	
pHGE-f	CACCTCGCTAACGGATTCACC
pHGE-r	CCAATACGCAAACCGCCTC
pMQ70-f	GCGTCACACTTTGCTATGCCATAGC
pMQ70-r	CTACTGCCGCCAGGCAAATTCTGTTT
pET28b-f	TAATACGACTCACTATAGGG
pET28b-r	TATGCTAGTTATTGCTCAG
pHGR01-f	CGTCAATTATTACCTCCACG
pHGR01-r	GTGCTGCAAGGCGATTAAG
P*mqsRA*-probe-f	CTATTAGCCAAATTTAACC
P*mqsRA* probe-r	CGCCTTTCAATGCAG
P*mqsRA* probe mutate-f	CTATTAGCCAAATTTAACCTGGATCACAGCTTTCTC
**Gene knockout**	
mqsA-up-f	CGGGGTACCCCTGTCCTACGCCAACCCC
mqsA-up-r	GCCCCCGCTAGCTGCGTACTGCCTGATAATGGC
mqsA-down-f	GCCCCCGCTAGCGGTTACAACTCCTTGAAA
mqsA-down-r	CCCAAGCTTCTGCCGTAGGCGGTGAGG
mqsR-up-f	CGGGGTACCCCTGTCCTACGCCAACCCC
mqsR-up-r	GCCCCCGCTAGCCCATGAGATGTCCAATAT
mqsR-down-f	GCCCCCGCTAGCGAAGTCTGCCCCAATGCCC
mqsR-down-r	CCCAAGCTTCTGCCGTAGGCGGTGAGG
pPS856-Gm-f	GCCCCCGCTAGCCGAATTAGCTTCAAAAGCGCTCTGA
pPS856-Gm-r	GCCCCCGCTAGCCGAATTGGGGATCTTGAAGTTCCT

delta *mqsA*-s-f	CTGCGACATCAAGGACCCG
delta *mqsA*-s-r	TGTATTTCGGGCGTCGTGAG
delta *mqsA*-l-f	TGGCTCGTTCCTTTATCACCTG
delta *mqsA*-l-r	CGCACTTCGCCGACCATCT
**qRT-PCR or RT-PCR**	
mqsR-RT-f	ATGGAAAAGAGAATGCCTCA
mqsA-RT-r	CTAGGCAGTACGCACTTCCTCAAAGAGT
mqsR-f	CTGCGGCAAGGCGAATC
mqsR-r	TCCTGCCAAACCCGGTGG
mqsA-f	CGACCTCGACCAGCGTGAA
mqsA-r	CCAGCAGCTTGAGCAGTTTC
algU-f	GATTCTCGGGTTGATCGTGCGG
algU-r	CCGTGTTGATGGCGATGCGGTA
PP_3288-f	CCGTGAATGGCTGGAAACCC
PP_3288-r	GCGCATCCACCAAGGCTCAT

Table S2. qRT-PCR results for the transcriptional levels of the *mqsR* and *mqsA* gene in the indicated strains. Strains used are indicated along with the cycle number (C_T) for each sample including that for the target genes as well as that of the house-keeping gene, *16S rRNA*, which was used to normalize the data (Pfaffl, 2001). Fold changes in the transcription of *mqsR* or *mqsA* in the mutant strains compared to the wild type strain were calculated as:

$$2\text{^-}(C_{T\ target\_mutant}-C_{T\ 16S\ rRNA\ mutant\_})/2\text{^-}(C_{T\ target\_wt}-C_{T\ 16S\ rRNA\_wt})$$

Gene	Strain	C_T value	Fold change
16S rRNA	KT2440	11.9 ± 0.16	
	Δ*mqsR*	11.7 ± 0.13	
	Δ*mqsA*	11.8 ± 0.13	
	Δ*mqsA*/pHEG	11.4 ± 0.15	
	Δ*mqsA*/pHEG-*mqsA*	11.3 ± 0.12	
mqsR	KT2440	23.9 ± 0.04	
	Δ*mqsA*	22.8 ± 0.01	-2.0 ± 0.10
	Δ*mqsA*/pHEG	24.5± 0.01	
	Δ*mqsA*/pHEG-*mqsA*	26.9± 0.01	-5.6± 0.19
mqsA	KT2440	23.9 ± 0.02	
	Δ*mqsR*	23.8 ± 0.17	1.0 ± 0.04

Table S3. Mass spectroscopy results of the co-purified protein with MqsA-His (refer to **Fig. 2A, line 4**). Peptide fragments identified by mass spectrometry analysis are highlighted in different colors, and their loci in MqsR protein are also shown.

Query ID	Peptide seq	Ions score	Identity score	E-value	Retention time(s)
23	VKALAAAR	25.77	16	0.005429	7.65
59	RTDFYK	22.1	17	0.017265	11.58
291	MPHCPLER	29.46	16	0.002605	11.42
347	RIRPTGAALK	36.43	13	0.000228	14.33
349	SMTSHIDHR	56.51	16	5.24889E-06	11.52
725	LSVVDDVLIVSFK	38.58	14	0.000201	14.58
869	ALGMDFPGMLEVITSLK	21.25	20	0.040869	12.73

Protein MqsR [*Pseudomonas putida* KT2440]

MqsR Protein: MEKRMPHCPLERVKALAAARRIRPTGAALKGAKALGMDFPGMLE
Identified: MPHCPLERVKALAAARRIRPTGAALKGAKALGMDFPGMLE
MqsR Protein: VITSLKRTDFYKSMTSHIDHRVWQDVYRPLTAIGYVYLKLSVVD
Identified: VITSLKRTDFYKSMTSHIDHR LSVVD
MqsR Protein: DVLIVSFKEL
Identified: DVLIVSFK

Supplementary Figure 1(this figure has been revised). The *mqsRA* loci in *P. putida* KT2440**.** DNA sequences from position -200 to position 701 on the sense-strand (from 5' to 3') relative to the translational start codon of *mqsR* are shown. The coding region of *mqsR* sequences is in blue font, and the coding region of *mqsA* is in orange font. The palindrome in the promoter region is marked with arrows, and the RBS regions of *mqsR* and *mqsA* are boxed. The primer pair *mqsR*-RT-f/*mqsA*-RT-r used for RT-PCR in the coding region of the two genes is underlined.

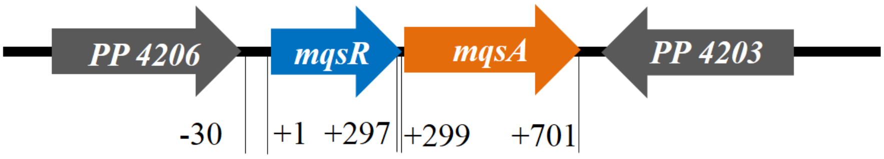

TCTGATCATGGGCGCGGTGGGGCTGCTGACGGCCCTGTATTTCGGGCGTC
GTGAGCCATTGGTGTTTGCGGCGGAGCCGATACCGGTCATGTGCGGCGAC
AAATGAGCCGGTAACGGCTGCGGGGCATTGGGGCAGACTTCCAGGTGGGG
GCTATTGGAATAACCTATTAGCCAAATTTAACCTGGAAGGTTAATTTCTC

ATGGAAAAGAGAATGCCTCATTGCCCGCTGGAGCGGGTAAAAGCCCTGGC
TGCGGCAAGGCGAATCCGCCCTACGGGTGCTGCATTGAAAGGCGCCAAGG
CGCTGGGAATGGATTTTCCGGGCATGCTTGAGGTGATCACCAGCTTGAAG
CGCACTGATTTCTACAAGAGCATGACCAGCCATATTGACCACCGGGTTTG
GCAGGATGTGTATCGCCCGCTTACAGCAATTGGGTACGTTTATTTGAAAT
TGTCCGTGGTAGACGACGTGCTCATCGTGTCTTTCAAGGAGTTGTAACCA
mqsR

TGAGATGTCCAATATGCGGCGGCTCGGAGCTTGCACCTGACATTCAGGGC
ATGCCCTATAGCTACAAAGGCGAGATGACAGTGATTCCTGAGGTAAGTGG
CGATTACTGCTCCGCATGTGGTGAATGTGTACTGAGTCATGATGAAGCCA
TGCGCGTCAGTCACTTGATGACGGCATTCGAGCGCCAGGTCAACGCAAAT
GTTGTGGATCCTTCCTTTATTGCCTCCATACGCAGAAAGTTCGACCTCGA
CCAGCGTGAAGCGGGGGAAATCTTCGGTGGTGGGGTCAATGCGTTCTCCC
GTTATGAAAACGGCAAGACCACCCCGCCAGTGGCGTTGGTGAAACTGCTC
AAGCTGCTGGATCGCCATCCAGAACTCTTTGAGGAAGTGCGTACTGCCTG
A
mqsA

Supplementary Figure 2. Gene *mqsR* and *mqsA* are co-transcribed. Total RNA was isolated from KT2440 at a turbidity of 1.0 at 600 nm. cDNA was synthesized and used as the template in PCR. Genomic DNA was used as positive control and total RNA treated with 5 U DNase I for 30 min was used in RT-PCR as negative control. M indicates DNA ladder.

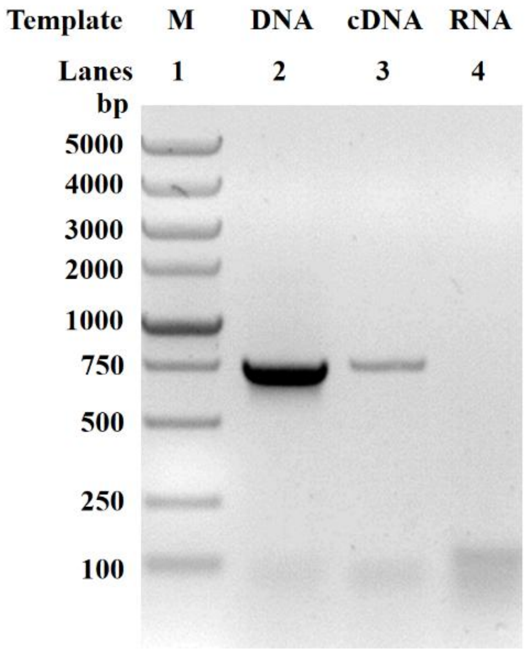

Supplementary Figure 3. Purification of MqsA**.** His-tagged MqsA was produced from pET28b-*mqsA*-His in *E. coli* BL21. After induction with 1 mM IPTG, the 15.51 kDa MqsA-His was induced (lane 3). The negative control was included when no IPTG was added (lane 2). SDS-PAGE showed that purified MqsA-His was obtained (lanes 4 and 5). Dimerization of MqsA was observed (lane 4), and the addition of the reducing agent dithiothreitol (DTT) greatly reduced the dimerization (lane 5).

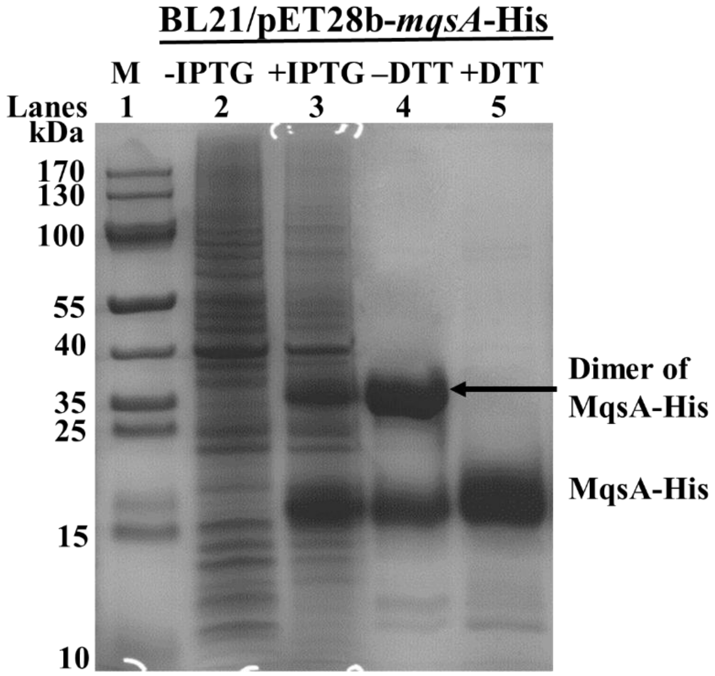

Supplementary Figure 4. Cell morphology of KT2440/pHGE, KT2440/pHGE-*mqsRA*, KT2440/pHGE-*mqsR* and KT2440/pHGE-*mqsA*. cells were collected at OD_{600}~1.0 after induced by 0.5 mM IPTG adding at OD_{600}~0.1. Three independent cultures were used and only one representative figure is showed here for each strain.

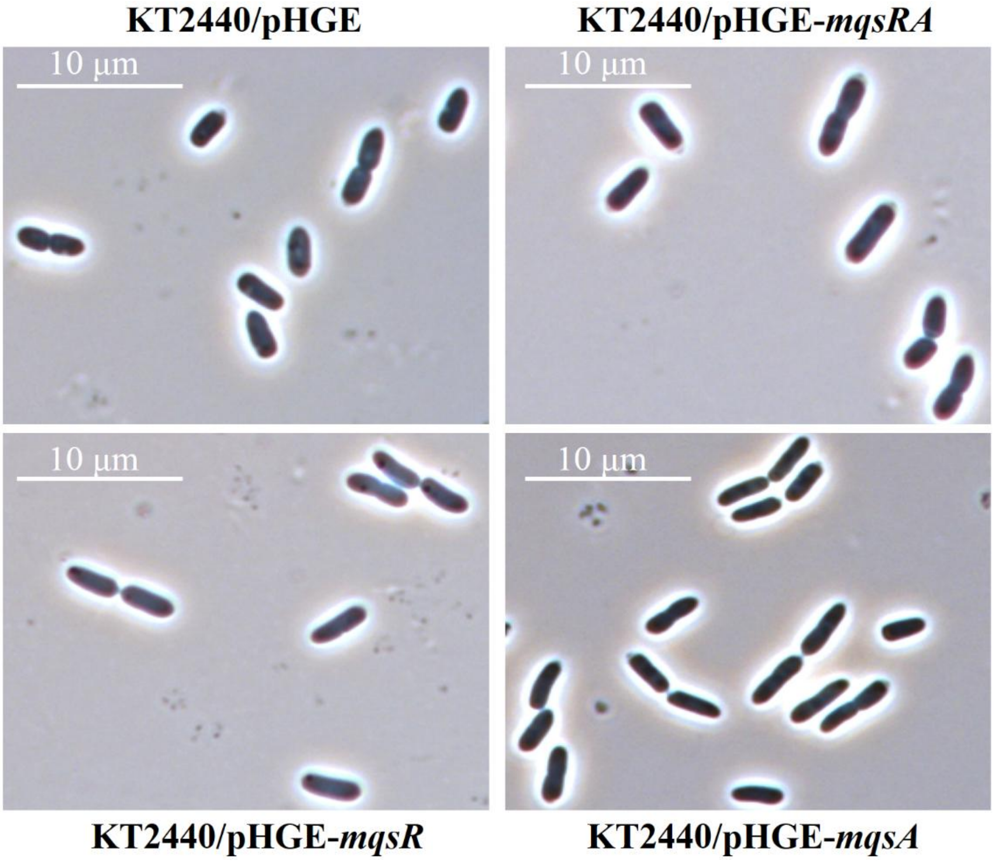

References:

Brown, B.L., Grigoriu, S., Kim, Y., Arruda, J.M., Davenport, A., Wood, T.K., et al. (2009). Three dimensional structure of the MqsR:MqsA complex: a novel TA pair comprised of a toxin homologous to RelE and an antitoxin with unique properties. *PLoS Pathog.* 5**,** e1000706. doi: 10.1371/journal.ppat.1000706.

Pfaffl, M.W. (2001). A new mathematical model for relative quantification in real-time RT-PCR. *Nucleic Acids Res.* 29. doi: 10.1093/nar/29.9.e45.
